# Approach to diagnosing and treating tears of the latissimus dorsi and teres major

**DOI:** 10.1016/j.xrrt.2025.05.015

**Published:** 2025-06-07

**Authors:** Kevin M. Lehane, Taylor Faust, Anthony A. Romeo, Brandon J. Erickson

**Affiliations:** a Department of Orthopedic Surgery, NYU Langone Health, New York, NY, USA; b Department of Research, Alabama College of Osteopathic Medicine, Dothan, AL, USA; c Department of Orthopedic Surgery, Duly Health and Care, Elmhurst, IL, USA; d Department of Orthopedic Surgery, Rothman Institute, New York, NY, USA

**Keywords:** Latissimus dorsi, Teres major, Throwing injuries, Repair, Shoulder, Overhead athlete

## Abstract

Latissimus dorsi and teres major tears are rare injuries encountered by orthopedic surgeons, most commonly seen in overhead throwing athletes. Due to their rarity and variable clinical presentations, these injuries can be challenging to diagnose and are often missed during initial evaluation. Enhancing the understanding of latissimus dorsi and teres major injuries is essential for developing optimal diagnostic and treatment strategies. This review offers a comprehensive overview of these uncommon injuries, including their indications, surgical and nonoperative approaches, and associated outcomes.

Tears of the latissimus dorsi (LD) and teres major (TM) tears are rare orthopedic, most frequently seen in overhead athletes; however, they are infrequently encountered by orthopedic surgeons.^18^ During the throwing motion, significant forces are exerted on the glenohumeral joint and its surrounding musculature.^21^ The resulting tears vary in type, with high level athletes frequently sustain avulsion type injuries.^42^ Due to the rarity of these injuries and a variety of exam findings, they are often difficult to diagnose and are frequently missed on initial presentation.^18^ Enhancing the understanding of LD/TM injuries are critical for developing optimal diagnostic and treatment strategies.

The LD and TM muscles primary functions are humeral adduction, extension, and internal rotation.^16^ These are important features specifically in overhead athletes due to their role in protecting the anterior aspect of the glenohumeral joint during the throwing motion. When there is a tear involving either the LD or the TM, the athlete's ability to execute the throwing motion is impaired and may be severely debilitating. Understanding the functions of the LD and TM are important due their involvement in various motions across different axes and planes, allowing for better identification of deficits during injuries.

## Latissismus dorsi anatomy and function

The LD is a large-fan shaped muscle with a broad origin, including the spinous processes of six lower thoracic vertebrae, the lumbar spine, thoracolumbar fascia, the most inferior three to four ribs, inferior angle of the scapula, and the iliac crest. It inserts into the floor of the intertubercular groove on the humerus after 90° of external rotation.^14^^,^^46^^,^^48^ From the extensor portion of the origin, fibers pass in multiple directions; upper fibers run horizontal, middle run obliquely upward, and lower fibers run vertically upward.^47^^,^^48^ The upper fibers insert more distally, while the inferior fibers insert are more proximally.^53^ The LD tendon expands to the deep fascia of the arm; it is innervated by the thoracodorsal nerve and is vascularized by the thoracodorsal branch of the subscapular artery.

The LD is a complex muscle with diverse mechanics, giving it the ability to function seamlessly and efficiently. It performs multiple functions, including shoulder extension, adduction, and internal rotation.^10^^,^^31^^,^^33^^,^^49^ The LD produces internal rotation of the humerus during acceleration and follows through with shoulder adduction during the late portion of the motion. The acceleration and follow through phases are most commonly seen in professional baseball players, contributing to their higher injury rates. The LD is initially activated at the point of maximum external rotation of the shoulder during late cocking phase.^32^ During the deceleration phase, the posterior shoulder musculature, particularly the TM and LD, eccentrically contract to slow the humerus, while resisting shoulder distraction and anterior subluxation forces.^20^ The LD also plays a role in arm internal rotation, extension of the spine, and contributes to overall core stabilization.^43^ These functions make the LD vital for various activities in the everyday movements, and especially important in overhead motion movements seen in sports like baseball, tennis, and gymnastics.

## Teres major anatomy and function

The TM is a small, rectangular shaped muscle, that originates from the posterior aspect of the inferior angle of the corner of the scapula and it inserts medially to intertubercular groove of the humerus.^30^ It is located inferior to the teres minor and travels anterior to the triceps long head before inserting on the humerus.^25^^,^^56^ The TM is located deep to the deltoid muscle, it forms the posterior wall of the axilla, and runs along the subscapularis and the LD muscles. Its blood supply comes from multiple arteries; the thoracodorsal, circumflex scapular, and the posterior humeral circumflex arteries.^23^ It is innervated by the lower subscapular nerve.^13^ The TM also plays a key of role in synergistic movement along with the LD.

The TM works in conjunction with the LD for several movements. It assists in shoulder extension by pulling the upper arm downward during overhead motions. It also aids in medial rotation of the arm, adduction of the arm, and shoulder joint stabilization of the shoulder joint.^25^

## Pathophysiology

Injury to the LD and TM can occur at various locations, from the tendon to the muscle belly.^3^^,^^6^^,^^9^^,^^44^^,^^45^ Four main types of LD ruptures have been described: isolated tendon injury or avulsion, combination of injuries (LD with pectoralis major, TM or rotator cuff), isolated myotendinous strain, and the least common intramuscular strain, also known as an LD costal tear.^2^^,^^22^ The most common injuries are grade 1 and 2 muscle belly strains in the LD and the TM.^1^ Tears in pitchers most commonly occur during the late cocking, early acceleration, and deceleration phases of the throwing cycle, when the muscles are most active.^10^ Injuries in nonpitchers are typically caused by forceful resisted shoulder adduction and flexion or by sudden forced extension or hyperabduction of shoulder.^4^^,^^9^^,^^26^^,^^28^^,^^38^^,^^39^

### Epidemiology

LD and TM injuries are uncommon in everyday activities; however, they have been reported in weight lifters and CrossFit athletes, a population that is more frequently encountered by the average orthopedic surgeon.^18^^,^^22^ The highest prevalence of LD tears is seen in throwing and overhead athletes, becoming more frequent with higher levels of play particularly at the professional level. Most cases in the literature have been reported in professional baseball pitchers, but they have also been described in water skiers, steer wrestlers, rock climbers, and track athletes.^9^^,^^15^^,^^18^^,^^28^^,^^38^^,^^39^^,^^42^ Isolated TM injuries can occur and have been documented in professional boxing, and hockey.^25^^,^^41^

### Risk factors

In overhead sports, several pertinent risk factors increase the likelihood of LD and TM injuries. Baseball pitchers are the most affected cohort.^19^ Factors associated with a higher incidence of tears in pitchers include increased innings pitched, a greater number of batters faced, and being a starting pitcher rather than a reliever.^10^ Proper throwing mechanics likely play a role in injury prevention, although no current literature has specifically linked them to LD and TM injuries. Some common risk factors in the general population include overuse and repetitive movements, muscle imbalances, poor posture, increased age, and lack of proper warm up.^54^ A better understanding of these risk factors and the implementation of preventive measures can help reduce the risks for muscle injuries.

## History and presentation

The history and physical exam are crucial for the clinical diagnosis of the LD and TM injuries. A comprehensive history should include details of prior injuries, or treatments (including injections), and periods of rest.^19^

A description of the pain's nature and the specific phase of throwing motion affected can be very informative in the diagnostic process. LD or TM injures may present acutely with a sudden onset of shoulder pain, sometimes accompanied by an audible pop.^22^ In addition, patients may report a prodromal posterior shoulder ache or discomfort.^2^ Patients may describe an immediate cessation of playing ability, an increasing level of pain, and even a burning sensation.^22^^,^^26^ In baseball, some pitchers can pinpoint the exact timing of pain; often occurring during ball release and follow through. Pain that begins in the late cocking or/early acceleration phases of the pitching cycle and persists through the deceleration phase is common, as this is when LD and TM are most active.^10^^,^^16^^,^^18^

### Physical exam

**Figure 1:**
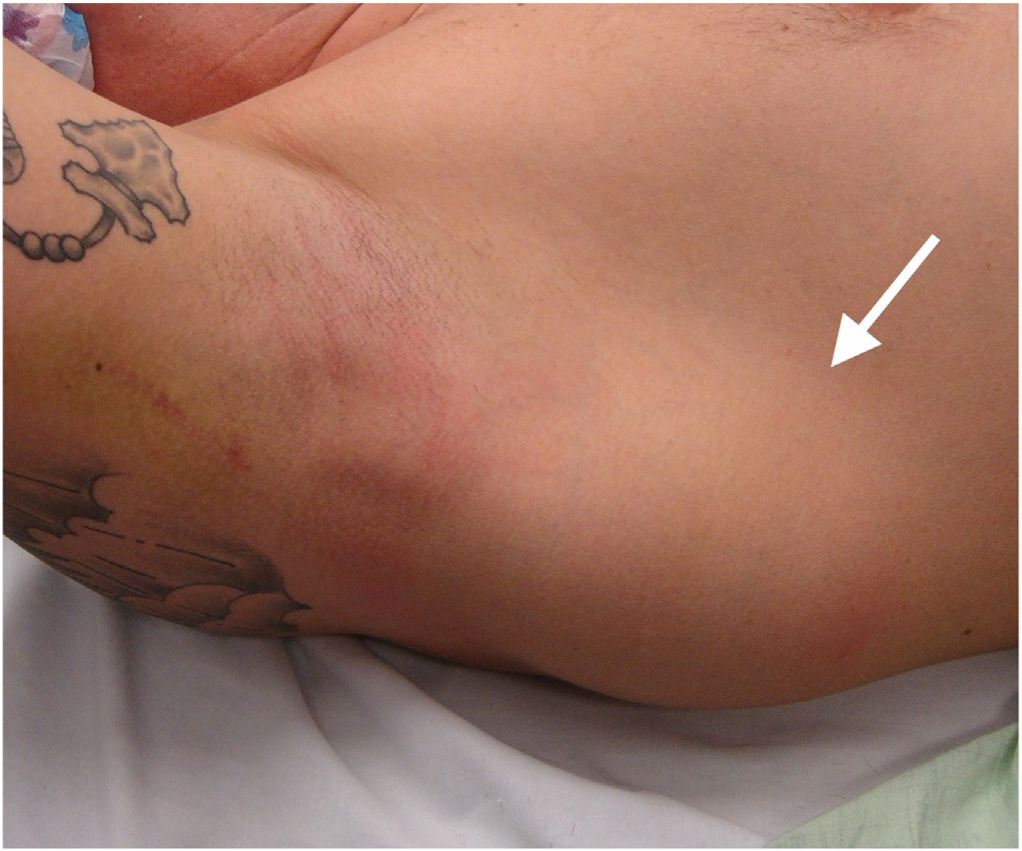
Physical exam findings of an athlete's right shoulder/axilla that demonstrates hematoma and swelling (*arrow*) which can be seen in a LD tear. *LD*, latissimus dorsi.

The physical exam for an LD and/or TM injury should be conducted with full exposure of the patient's shoulder and scapula. The first step is inspection, as acute, significant tears can present with edema, ecchymosis, and deformity.^22^^,^^35^ Tears may also present with a large hematoma in the posterior axilla (Fig. 1). In addition, atrophy may be observed, depending on the time elapsed from injury to encounter with the provider. There may also be visible defects along with compensatory hypertrophy or prominence of surrounding muscles in the region.^50^ Isolated TM tears can feature swelling and hematoma at the scapula following the injury.^11^

Pain and deficits during active range of motion (ROM) of the shoulder in flexion, abduction, external rotation, and internal rotation may be present.^44^^,^^53^^,^^55^ Weakness is often noticeable in extension, adduction, and internal rotation.^9^^,^^15^^,^^35^^,^^45^ If the LD or TM is retracted, there may be loss of the posterior axillary fold. Palpation of the entire LD/TM from origin to insertion is essential, assessing for tenderness, side to side differences in muscle bulk, tension, etc. In cases of an avulsion injury, palpation may reveal a defect at the insertion site.^36^ The belly-press and lift-off tests, typically used in rotator cuff evaluation, can produce pain or weakness in LD and TM injuries.^43^^,^^44^

A thorough neurovascular exam is essential to document any specific nerve injuries. The radial nerve specifically can be compromised in traumatic incidents, due to its close proximity to the LD and TM. This may present with wrist drop or paresthesias in the forearm.^19^

### Imaging

Imaging studies are essential for confirming LD and TM tears determining the extent of the injury. Initial diagnosis begins with a full shoulder radiographic shoulder series, including anteroposterior, axillary, scapular Y, and Grashey views. These images help identify bony avulsion injuries and rule out other potential causes of shoulder pain.^38^

**Figure 2:**
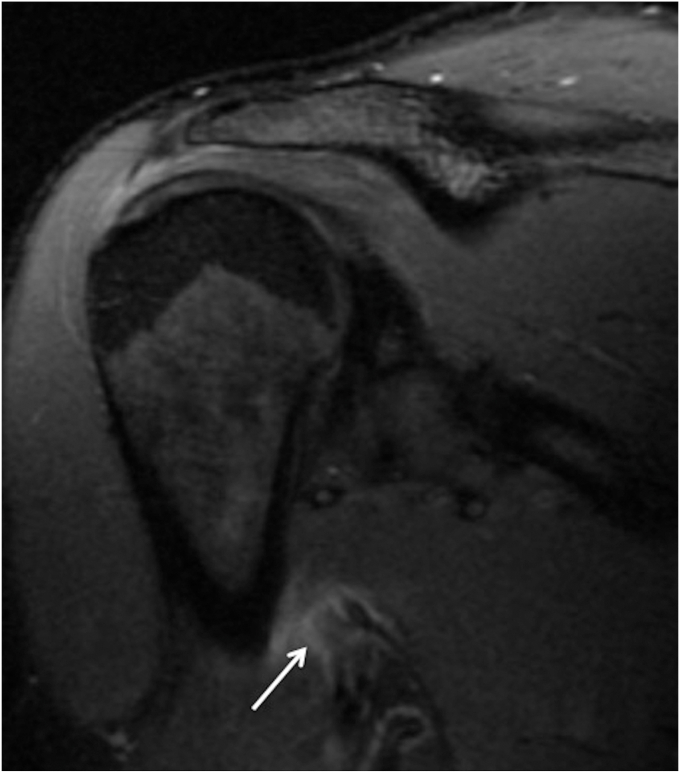
Coronal MRI of grade IIIA latissimus dorsi/teres major tear with retraction of tear and surrounding edema (*Arrow*). *MRI*, magnetic resonance imaging.

Diagnosis of LD and TM injuries is most frequently made and confirmed using magnetic resonance imaging (MRI) and ultrasound (US). In the acute setting, a noncontrast MRI is preferred.^14^^,^^19^^,^^44^ MRI can reveal discontinuity of the tendon or muscle, allowing for a quantification of distance of retraction in tear injuries. In LD/TM tears there can be increased fluid signal intensity surrounding the injury can permeate inferiorly through the muscle, enhancing visualization (Fig. 2).^17^

The MRI should include a dedicated shoulder series as well as a series of the chest wall, as the standard MRI views can miss LD and TM injuries.^53^ Using MRI views of the upper thorax and shoulder girdle improves detection of the injury. This may require specific guidance of the radiology technician to ensure the extent of the muscle is included in the field. Expanding the view will encompass both the LD and TM origin and insertion, allowing allow for a comprehensive assessment of the injuri's extent.^17^^,^^34^^,^^40^

Due to the rising incidence and potential for long-term functional impairment from LD and TM tears, it is important to develop a grading system to improve communication between physicians, coaches, training staff, and rehabilitation teams for optimal athlete management to best manage the athletes.^16^ An MRI classification system has been introduced to better define LD and TM tears, correlating severity with return to sports (RTS) outcomes and performance upon RTS. It also offers predictors of nonoperative management failure, enabling timely surgical intervention for LD and TM tears.^36^

**Figure 3:**
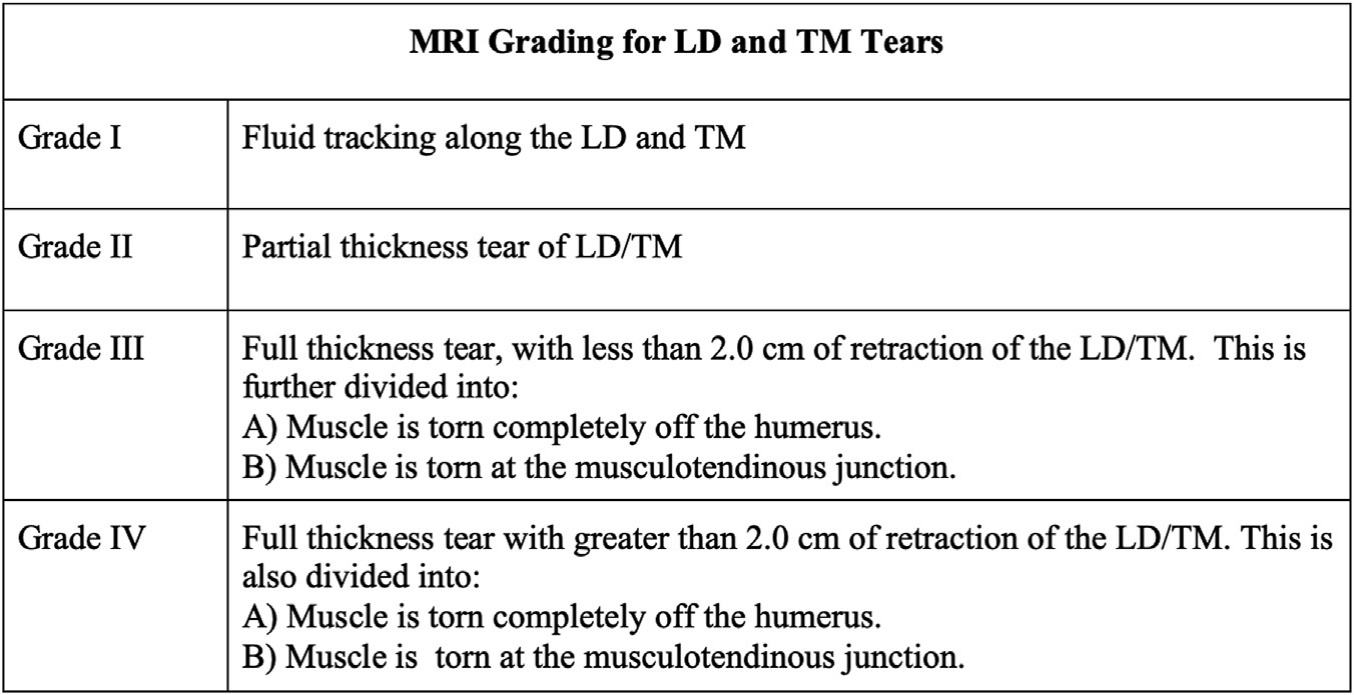
MRI grading of LD and TM tears based on Erickson et al.^17^*MRI*, magnetic resonance imaging; *LD*, latissimus dorsi; *TM*, teres major.

**Figure 4:**
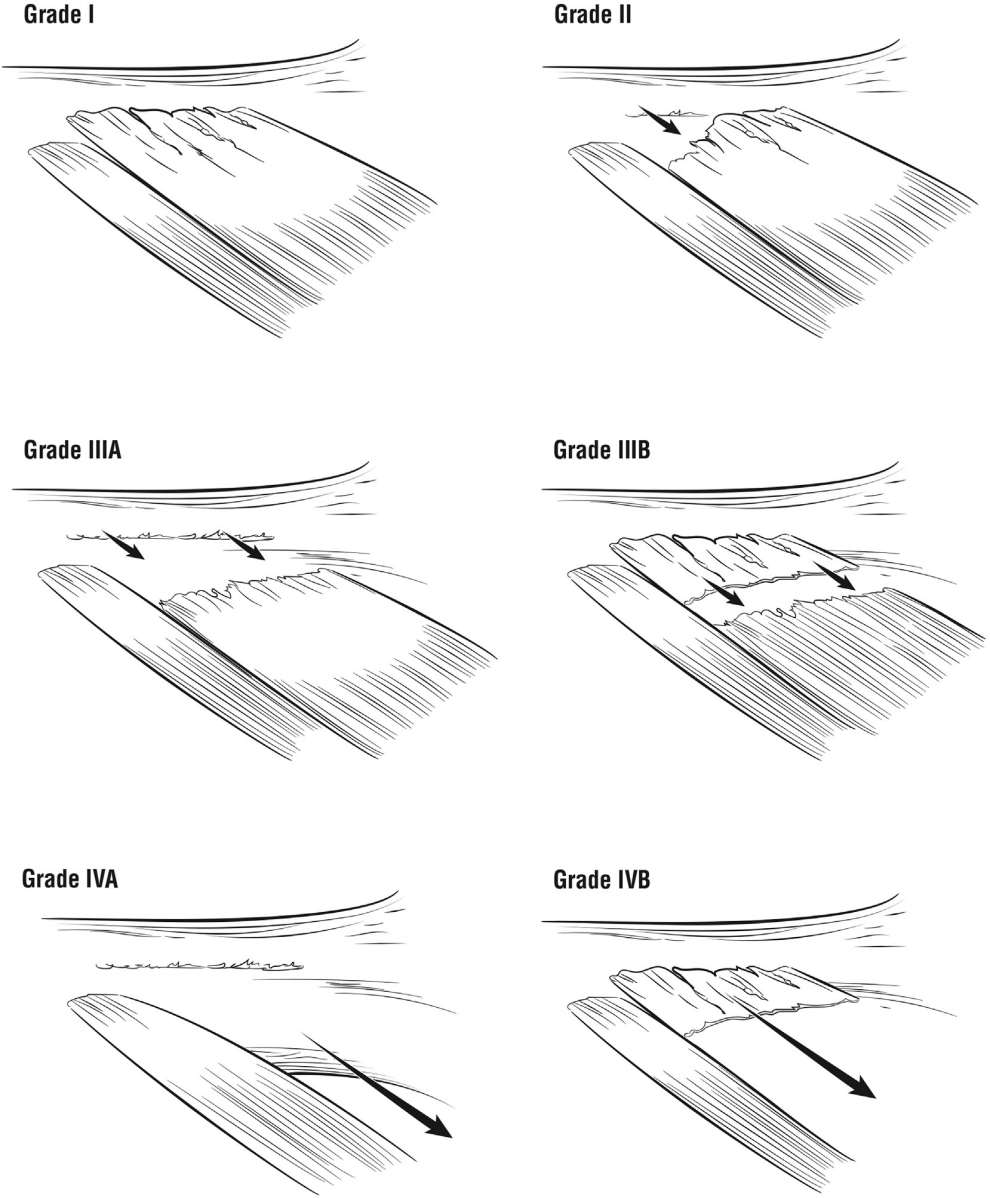
Provides a depiction of grade I tears through grade IV for a latissimus dorsi/teres major tear.

The novel grading system by Erickson et al classifies LD and TM tears into four levels based on MRI findings (Figs. 3 and 4). The grading system is critical for determining the optimal initial management plan. Lower grade tears (I and II) can typically be managed nonoperatively, while high grade tears (III and IV) are more likely to require surgical intervention. In the study, high grade tears (III and IV) were initially treated nonoperatively and had a failure rate of 83.3%. However, athletes who failed nonoperative management were able to successfully RTS after delayed surgical repair.^17^

US can also be useful in the diagnosis of LD/TM tears.^25^^,^^47^ US imagery utilizes a posterior axillary approach for diagnostics of a possible tear.^9^ Common findings on US include the following: hypoechogenicity or hyperechogenicity and swelling within the muscle, myofibril disruption, and possibly tendon retraction.^2^^,^^47^ US is also valuable for marking the site of tendon retraction tendon and aid in surgical planning by guiding the incision.

## Treatment

Treatment of LD and TM injuries varies based on the extent of the injury, grading, and the patient's clinical history. Management approaches include nonsurgical and surgical interventions, each with specific indications and protocols.

### Nonoperative

Nonsurgical management is the first-line treatment for Grade I and II injuries, according to the current literature.^17^^,^^35^ Conservative treatment involves a multimodal approach, including: rest, anti-inflammatory medications, cryotherapy, red laser therapy, injections (steroids or platelet-rich plasma [PRP]) and rehabilitation techniques that focused on gradual strengthening and return to activity. The management protocol includes an initial rest period and sling immobilization to address the acute inflammation with a shutdown period lasting 1-28 days. For grade II injuries, a 5 day course of methylprednisolone is recommended before transitioning to oral non-steroidal anti-inflammatory drugs.^19^^,^^35^ Core and lower body strengthening exercises should be introduced during the later rehabilitation stages to offset the shoulder stress and prevent reinjury.^14^ Once the patient is pain free, gentle ROM experiences can begin followed by physical therapy and rehabilitation programs.^50^ Pendulums exercises should be introduced early to prevent any stiffness during the initial period of rest.^19^

Other nonoperative modalities to consider include: steroid or PRP injections.^19^ These injections provide a direct anti-inflammatory effect, beneficial for the acute phase of injury.^19^ PRP injections in multiple clinical case series have demonstrated evidence for faster healing, and decreased inflammation in various muscle strain injuries.^19^ Although beneficial for LD and TM injuries, there is currently a need for more data specifically addressing its efficacy for these injuries.

Rehabilitation is a progressive process that begins with active-assisted ROM and/or passive ROM and strengthening exercises.^5^ Gradually, strengthening programs designed to restore full function. Rehabilitation should culminate in sport-specific exercises, focusing on a RTS. In baseball players, a throwing program is essential to gradually reintroduce the LD and TM to the stresses of the throwing motion.^29^ If the athlete experiences pain during the throwing program, they should immediately cease any activity and restart with a period of rest and recovery. In many throwing athletes recovering from LD and TM injuries will have the patients participate in a return to throw program as described by Sgroi et al. Further details regarding the rehabilitation process are discussed in the subsequent sections.

### Outcomes

Nonoperative treatment of LD and TM injuries has demonstrated successful outcomes in literature, particularly in grade I and II tears. Nagda et al reported 15/16 patients successfully returned to the same level or high level of play following nonoperative treatment. Of these, nine players without avulsion injuries returned to play, missing an average of 82.4 days. Players with avulsion injuries experienced season ending setbacks and reduced performance after nonoperative treatment but were eventually able to return to play. Of these, 13% athletes suffered recurrent injuries.^44^ Erickson et al studied 107 pitchers with LD or TM injury and found that 75% returned to play rate, which was comparable to those in the operative cohort. The pitchers who were able to return to full participation took an average 170 days from injury. However, those treated nonoperatively noted a decline in several performance statistics, including a higher walks plus hits per inning pitched and had a fewer number of games played per year.^16^ Additional reports described 8 out of 10 pitchers returning to professional of level, with performance levels comparable to preinjury.^53^ Another study mentioned isolated TM injuries in pitchers; one had an avulsion injury and was able to RTS after 6 months from injury and another patient with isolated grade II TM strain was able to RTS at 9 weeks.^35^

### Surgical management

Surgical management is an effective treatment option for LD and TM tears with retraction, grade III and IV tears, and cases after failure of conservative treatment.^10^^,^^14^, ^15^, ^16^^,^^35^^,^^42^^,^^53^ With all surgery, it is important to highlight and consider careful complication of repair, due to the location of the muscles. Knowledge of the neurovascular region is vital, as the radial nerve, axillary nerve, and posterior brachial cutaneous nerve are in the surrounding region. Surgeons must account for these structures to avoid nerve damage and significant complication of the procedure.^19^

### Indications for surgery

The indications for surgical repair of LD/TM tears in the literature included are those who have failed a trial of nonoperative treatment in low grade injuries, those with grade III and IV tears. Grade III and IV tears involve a full-thickness muscle tear, with grade III having less than 2.0 cm of retraction and grade IV featuring greater than 2.0 cm of retraction.^17^ Further refinement and classification of surgical indications, beyond MRI findings is essential to assist surgeons in determining the appropriate decision and timing for repair, surgical approaches, and procedural techniques necessary for effective surgical intervention.^17^

### Surgical approach

The importance of patient positioning and surgical approach in addressing LD and TM injuries lies in optimizing exposure and access to ensure the success of surgical intervention. Patient positioning and approaches are chosen based on specific characteristics of the injury. Patient positioning options include beach chair, lateral, or supine positions, with the specific approach options depending on the position has been shown to have varying opinions. Despite various techniques described in the literature, no single optimal approach or position for these injuries has been properly defined. Each position provides unique advantages, but it is ultimately up to the surgeon's preference based on experience and type of tear in selecting the position.^12^^,^^15^^,^^26^, ^27^, ^28^^,^^38^^,^^39^^,^^56^

Various surgical approaches have been described in the current literature. Ellman et al described a two incision method with an anterior axillary incision in the subpectoral area approximately 1.0 cm medial to the bicipital groove, parallel with the axillary fold. A secondary posterior axillary incision is used to facilitate the safe retrieval of the retracted tendon and/or release adhesions that may have formed in subacute or chronic injuries.^15^ Henry and Scerpella et al used an axillary incision with the patient supine and the arm abducted for tendon retrieval. A second anterolateral arm incision for drilling of transosseous tunnels.^27^ Livesey et al utilized the beach-chair position, with the arm abducted. The two incisions included 1 over the posterior axillary fold for tendon retrieval and a second one for the deltopectoral incision for fixation.^39^

**Figure 5:**
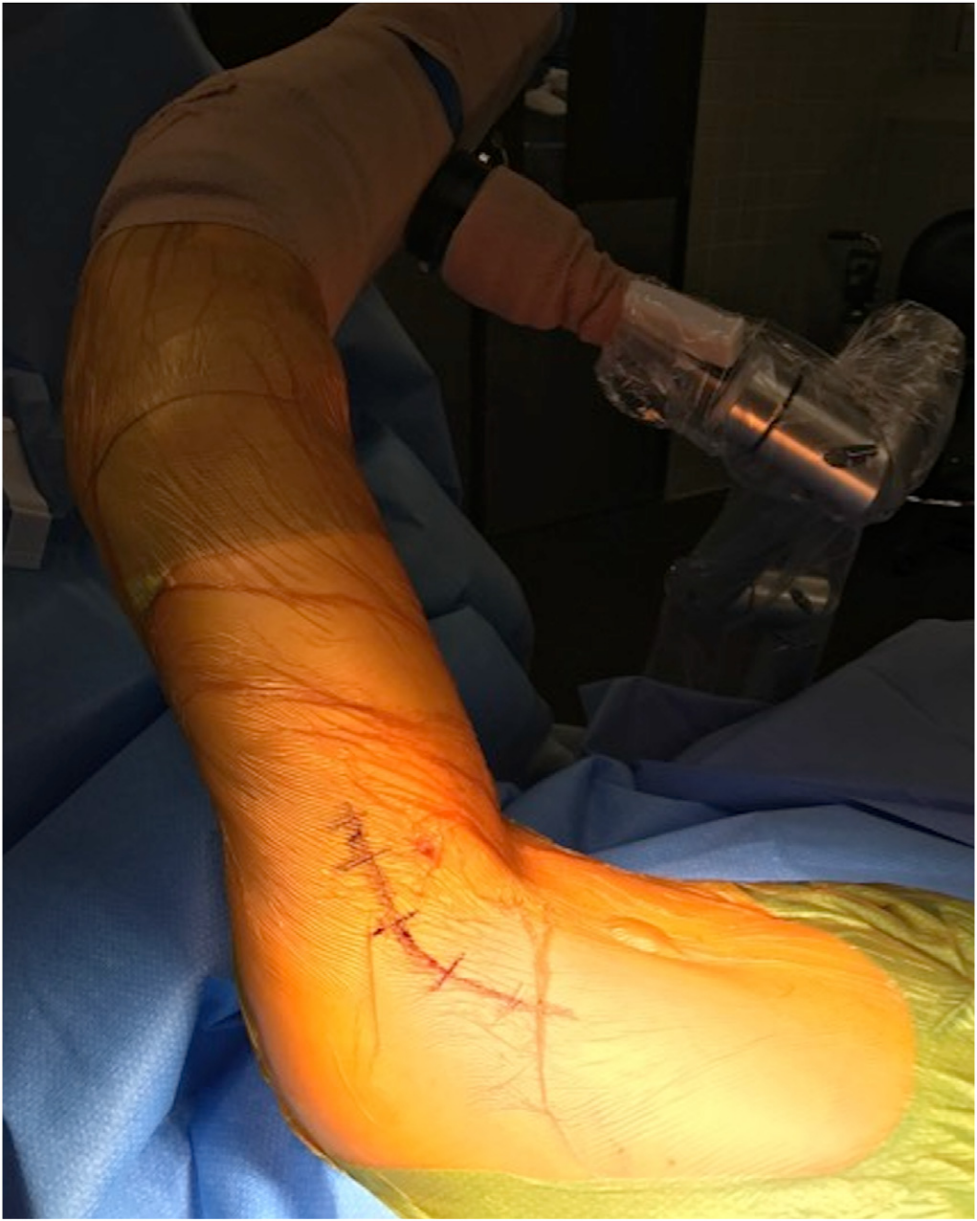
A patient positioned in the lateral decubitus position with arm in slight flexion, abduction and internal rotation, with a posterior axillary incision for LD/TM repair. *LD*, latissimus dorsi; *TM*, teres major.

**Figure 6:**
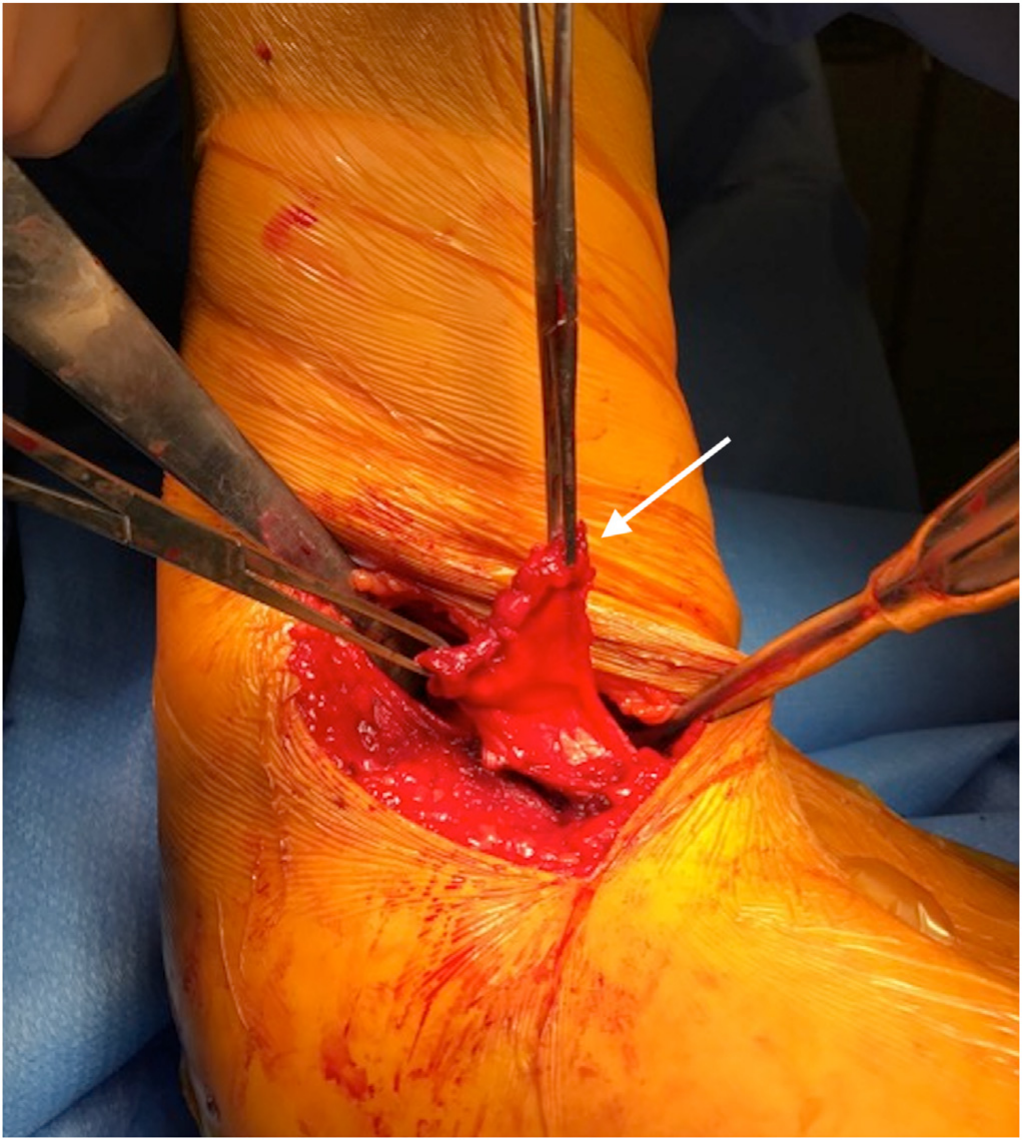
A posterior axillary approach demonstrating adequate exposure of the LD tendon retrieval (*Arrow*). *LD*, latissimus dorsi.

**Figure 7:**
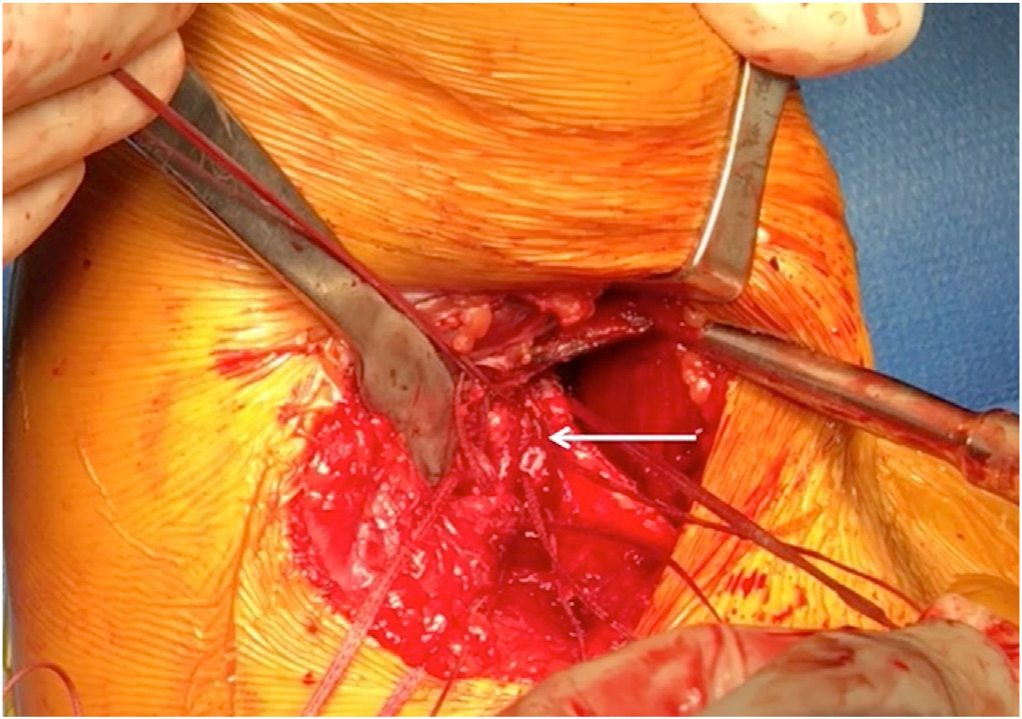
A posterior axillary approach demonstrating adequate exposure for fixation of the LD tendon into the humerus (*Arrow*). *LD*, latissimus dorsi.

While dual incision techniques are commonly utilized, single incision approaches have also been described. The most common approach utilized is a single posterior axillary incision, with the shoulder forward flexed, internal rotated, and slightly abducted with the patient in lateral decubitus position.^6^^,^^34^ An arthroscopy arm holder, such as the TRIMANO (Arthrex, Naples, FL, USA), can be very useful in positioning of the arm (Fig. 5). The significance of patient positioning and surgical approach is vital to the overall success of the LD and TM repair. It offers proper visualization of the surgical site, facilitates ease in tendon retrieval, and achieving secure fixation of the tendon to the bone are factors that are essential for favorable outcomes (Figs. 6 and 7).

Fixation techniques for LD and TM repairs include bone tunnels, suture anchors with tenodesis, suture anchors only, interference screws, fiber wire cerclage, and suture repair within musculotendinous junction.^12^^,^^24^^,^^26^, ^27^, ^28^^,^^37^, ^38^, ^39^^,^^56^ The tendon is usually secured with a Krackow stitch using a large nonabsorbable suture.^15^^,^^24^ While numerous surgical techniques exist, additional studies are required to determine whether there is an optimal approach or fixation type.

### Outcomes

Most of the existing operative outcome literature consists of case series, many of which are authored by Erickson et al. One of their case series examined the return to play (RTS) for 16 baseball pitchers who underwent operative treatment. Among professional baseball players who were more than 1 year out from their injury or surgery date, 100% were able to return to their prior level of play. Five patients were initially managed nonoperatively, but required surgical intervention. Time to RTS for operative group was approximately 10 months.^17^ Another case series by Erickson et al reported on 11 patients who underwent surgical repair of the LD/TM and found that all 11 professional baseball pitchers returned to their prior level of play, and saw significant improvements in visual analog scale scores, and shoulder outcome scores.^18^ A third study by Erickson et al followed 7 professional pitchers who underwent operative fixation of LD and/or TM tears, evaluating RTS and performance postoperatively. There was a 75% RTS reported with no significant differences found in earned run average, innings pitched, games played, or walks plus hits per inning pitched from before injury compared to preinjury performance.^16^

### Postoperative rehabilitation

Following surgery repair, the patient is placed in an immobilization sling with a 4-inch abduction pillow. The shoulder is kept in internal rotation and slight abduction for 4-6 weeks. The sling will prevent stressing the repair, as well as limiting adduction or excessive abduction.^19^

In the initial stages of physical therapy, postoperative movement will include gentle shoulder pendulum exercises as well as elbow/hand/wrist ROM once or twice per day. Passive ROM exercises can begin at 2 weeks following surgery. This will progress with passive exercises becoming active ROM exercises, until full ROM is achieved, which can take between 8 and 12 weeks. Once the patient is pain free and regains full ROM and strength they can begin strengthening exercises. Baseball specific throwing programs can begin at 12 weeks to 16 weeks postoperatively. RTS at 6 months is the earliest mentioned, with full recovery being defined in the literature between 9 and 12 months.^19^ The importance of shoulder therapy and complete shoulder ROM, internal and external rotation, as well as dysfunctional hip ROM has been shown to be a risk factor for shoulder injuries in pitchers.^7^^,^^8^^,^^57^

### Complications nonsurgical and surgical management

Surgical complications can include a variety of issues, current literature mentions complications that includes damage to adjacent radial nerve, axillary nerve, and neurovascular pedicles to these muscles.^46^^,^^52^ Time of injury can also influence complications such as the distance of the tendon retraction and the amount of scar tissue adhesions that need to be released for mobilization. Chronic tears may lead to fatty atrophy of the muscle and degradation of tendon integrity, which may necessitate the use of graft augmentation to strengthen the tendon.^50^^,^^51^

## Conclusion

Due to the uncommon occurrences of LD and/or TM tears, there is a pressing need for further research, as the current level of knowledge remains limited and lacks algorithmic and formulaic treatment plans. The existing evidence offers numerous options and guidance about the diagnosis and treatment for these injuries, but a greater data collection is necessary to enhance classifications and grading systems. This will help identify stronger predictors for more effective diagnostics and treatment. This review outlines key features that can guide a nuanced approach to managing these rare injuries. By presenting a comprehensive and deep framework for diagnosis, treatment, and outcome optimization, it offers a foundation that will help develop more effective interventions for patients affected by LD and TM tears.

Further delineation of nonsurgical and surgical approaches will be critical aspects for the management of these injuries. Exploring whether a combination of both modalities could yield improved patient outcomes A multidisciplinary collaboration between surgeons, rehabilitation specialists, athletic trainers, and researchers will be essential for refining comprehensive management strategies for these injuries.

As research into LD and TM2 tears continues to evolve more diverse perspectives and variable outcome analysis will be necessary. Expanding the data pool will not only help identify best practices but define the optimal treatment algorithms to improve long-term functional outcomes for athletes and patients with these rare injuries.

## Disclaimers:

Funding: No funding was disclosed by the authors.

Conflicts of interest: Anthony A. Romeo, MD, receives royalties from Arthrex Inc., research support, and is a paid consultant; from Saunders/Mosby-Elsevier and SLAC Inc., he receives publishing royalties and financial or material support; from AANA and MLB, he receives other financial or material support; from Paragen Technologies, he receives research support and stock or stock options. Brandon J. Erickson, MD, is a paid consultant for Arthrex; receives research support from Arthrex, Depuy, Linvatex, Smith & Nephew, and Stryker; and serves on committees for the AAOS, AOSSM, and ASES. The other authors, their immediate families, and any research foundation with which they are affiliated have not received any financial payments or other benefits from any commercial entity related to the subject of this article.
